# How modern treatments have modified the role of surgery in pediatric low-grade glioma

**DOI:** 10.1007/s00381-024-06412-w

**Published:** 2024-04-27

**Authors:** Scott Boop, Nir Shimony, Frederick Boop

**Affiliations:** 1 Department of Neurological Surgery, University of Washington, Seattle, WA USA; 2 Department of Surgery, St. Jude Children’s Research Hospital, Memphis, TN USA; 3 Le Bonheur Neuroscience Institute, LeBonheur Children’s Hospital, Memphis, TN USA; 4 Department of Neurological Surgery, University of Tennessee Health Science Center, Memphis, TN USA; 5 Department of Neurosurgery, Johns Hopkins University, Baltimore, MD USA; 6 Semmes-Murphey Clinic, Memphis, TN USA; 7 Global Program, St. Jude Children’s Research Hospital, Memphis, TN USA

**Keywords:** Pediatric, Glioma, Low-grade, Gross total resection, Radiation therapy, Targeted therapy, Immunotherapy, Ultrasound

## Abstract

Low-grade gliomas are the most common brain tumor of childhood, and complete resection offers a high likelihood of cure. However, in many instances, tumors may not be surgically accessible without substantial morbidity, particularly in regard to gliomas arising from the optic or hypothalamic regions, as well as the brainstem. When gross total resection is not feasible, alternative treatment strategies must be considered. While conventional chemotherapy and radiation therapy have long been the backbone of adjuvant therapy for low-grade glioma, emerging techniques and technologies are rapidly changing the landscape of care for patients with this disease. This article seeks to review the current and emerging modalities of treatment for pediatric low-grade glioma.

## Introduction

### Epidemiology

Low-grade gliomas (LGG) are the most common brain tumors in childhood, comprising 35% of all pediatric tumors [1]. Given that complete resection offers a high likelihood of cure and near-total resection is still associated with a reasonable outcome, caring for patients with LGG can be very rewarding. In one of the largest observational studies of childhood brain tumors, Wisoff et al. demonstrated that, regardless of subtype, as many as 80% of children with low-grade glioma can expect to survive a decade [2]. Throughout our first century of neurosurgery as a specialty, complete resection of LGGs has offered the best chance for cure. In this article, the authors provide an overview of emerging technologies which are being studied as treatment alternatives for LGG when surgery is not curative. It should be noted that, although there have been numerous advances in both diagnostics and therapeutics for LGG, at present, gross total resection still offers our patients the best opportunity for a cure. That said, there are certain situations in which these tumors are not amenable to complete resection, for which we must rely on other treatment modalities.

### Radiology

Advances in neuroimaging in the hands of an experienced neuroradiologist will allow pre-operative diagnosis of LGG in over 90% of cases. Furthermore, contemporary MRI sequences will allow the neurosurgeon to determine whether the tumor displaces normal brain structures versus infiltrating them. Functional neuroimaging can also determine the interface between the LGG and adjacent speech, motor, or sensory cortex. Tractography can determine which tumors are infiltrative versus confluent as well as where functional connections are displaced. High-field intraoperative MRI has also allowed us to double our likelihood of achieving a gross total resection or maximal safe resection with less morbidity [3, 4].

### Molecular classification

In 2021, the fifth edition of the WHO classification of brain tumors was based on the genetic alterations common to various central nervous system tumors. This classification schema classifies gliomas, glioneuronal tumors, and neuronal tumors into six families, three of which include pediatric low-grade gliomas and glioneuronal tumors (pLGG/GNTs). Included in the new classification are six newly defined pLGG/GNTs. These include “diffuse astrocytoma, MYB or MYBL1-altered”; “polymorphous low-grade neuroepithelial tumor of the young (PLNTY)”; “diffuse low-grade glioma-MAPK altered”; “diffuse glioneuronal tumor with oligodendroglioma-like features and nuclear clusters (DGONC)”; “myxoid glioneuronal tumor (MGT)”; and “multi-nodular and vacuolating neuronal tumor (MVNT).” The article by Bale and Rosenblum is an excellent review of the new classification of pLGG/GNTs of childhood [5].

### New radiation modalities

Over the decades, radiation therapy has proven effective in controlling the progression of low-grade gliomas. In the early years, the application of photon therapy through two opposing beam fields offered reasonable tumor control but at the expense of radiation side effects extending to large areas of normal brain and surrounding tissues. Given the longevity associated with most low-grade tumors, malignant degeneration and secondary neoplasia became a significant concern given patients’ long-term survival. With MRI-directed three-dimensional conformal radiotherapy, more precise delivery of photons has allowed improved sparing of normal tissues with notable improvements in quality of life and with less likelihood of secondary neoplasia. More recently, proton therapy has offered the advantage of the Bragg peak effect, and, with modern computer modeling, has substantially improved radiation delivery to the target with very little energy deposition along its trajectory. Carbon ion therapy is an emerging technology which, like proton therapy, has the advantage of an improved Bragg peak. The energy deposition with carbon ions is a steeper curve than that of protons. This modality is under investigation at over a dozen centers and for a variety of cancers [6]. Stereotactic brachytherapy is the local application of radiation-emitting isotope seeds implanted into the tumor. Historically, this treatment modality has a proven track record but has fallen into disuse in the modern era given the improved ability to deliver radiation modalities with greater precision through non-surgical means [7].

## Focused ultrasound

Another emerging modality is the use of high-frequency focused ultrasound for the treatment of glioma. This application is interesting in that it can provide both an additional ablative modality, as well as be capable of opening the blood-brain barrier (BBB) to enhance chemotherapeutic delivery. Ultrasonic waves are sound waves with a frequency of > 20,000 Hz. They have been in use for decades as a non-invasive imaging modality. Traditional use of intraoperative ultrasound has been as an adjunct for real-time intraoperative tumor visualization in order to guide resection [8].

Recent advances in high-intensity ultrasound combined with stereotactic techniques and transcranial magnetic resonance guidance have opened new avenues for the use of magnetic resonance-guided focused ultrasound (MRgFUS). This high-intensity ultrasound modality uses 650 to 750 kHz frequency to induce thermal tissue ablation, providing a selective focus of tissue temperature increase up to 65 °C [9]. This modality has been widely used in neurosurgery to create spatially precise ablations for multiple pathologies, including thalamotomy for essential tremor and tremor-dominant Parkinson’s disease [10, 11].

Early clinical studies of MRgFUS thermo-ablation for CNS tumors have predominantly focused on high-grade glioma, and no studies have specifically looked at pediatric patients. Multiple phase I trials are actively assessing this modality for CNS tumor ablation (NCT01473485, NCT00147056, NCT01698437, and NCT04559685), though so far no completed randomized controlled trial data is available and results are limited to preliminary findings and proof of concept case series. These have shown promising results, though limitations exist with this modality, given small treatment areas of effect and time required to ablate larger volumes [12, 13]. As such, more work is needed to explore this modality for CNS tumor treatment. Currently, the use of this modality in glioma treatment has been limited to recurrent or residual tumors that are not amenable to resection or conventional radiation therapy [14].

Another potentially exciting use of ultrasound technology for LGG therapeutics has been demonstrated by the ability of low-intensity FUS to disrupt the BBB. This technique does not cause thermo-ablation or irreversible tissue damage. Instead, circulating microbubbles are combined with low-intensity ultrasound bursts, concentrating the US effects on the vasculature and resulting in temporary local disruption of the BBB, increasing the permeability of large molecules [8]. Animal models using this modality have shown BBB disruption for up to 24 h after a single treatment, depending on the size of administered microbubble contrast agents [15]. Further preclinical studies have investigated this modality in combination with multiple chemotherapeutic agents, including temozolamide, bevacizumab, doxorubicin, and carboplatin, to enhance drug delivery to difficult-to-treat CNS tumors [9]. In addition, this approach has been used to deliver viral and T cell-directed therapy as well [16, 17]. Currently, multiple trials are ongoing to assess FUS as a means of increasing chemotherapeutic efficacy in CNS tumors, primarily glioblastoma in adults [8]. Although no studies are currently exploring this modality for its potential in pLGG, there are ongoing trials to assess its utility for pediatric patients with diffuse midline glioma/DIPG (NCT05123534 and NCT04804709) [8]. Another potential role for FUS-mediated BBB disruption is in the form of enhancing biomarker detection with liquid biopsy, though no clinical studies have published results for this to date [18].

## Laser interstitial thermal therapy

Laser interstitial thermal therapy (LITT) is one of the recent surgical techniques that is being used for the treatment of brain tumors. This modality uses real-time MRI thermographic guidance and targeted thermal energy delivered via laser fiberoptic probes surgically implanted into a structure. This allows for the delivery of focused thermal energy such that degradation of the cell membrane in the core tissue occurs. The approach is minimally invasive and, when the surgeon obtains a stereotactic needle biopsy of the tumor, he or she can introduce the laser ablation catheter through the same trajectory.

The first reported case using LITT in a pediatric patient was in 2011 [19]. Although more than a decade has passed since this report, the literature concerning the treatment of pediatric brain tumors using LITT remains relatively scarce in comparison to the adult population. The literature in adults mainly relates to HGG and metastatic disease, as well as radiation necrosis. Preliminarily, these reports show promising results regarding disease control with relatively low perioperative morbidity [20, 21]. In 2020, a multicenter retrospective study regarding the use of LITT in pediatric brain tumors showed promising results for pLGG, demonstrating tumor volume reduction [20]. The question remains whether thermal ablation alters the oncologic course of the disease [22]. LITT has been shown to reduce the length of hospital stay and improve patient satisfaction in several studies, but the evidence for its benefit over conventional craniotomy regarding progression-free survival is lacking [23].

For HGG, it has been shown that LITT has the additional benefit of disrupting the BBB, thus allowing for better delivery of chemotherapy (e.g., doxorubicin), with increased overall survival for patients treated [24]. One of the interesting emerging uses of LITT is as a substitute for other forms of focal energy delivery such as re-irradiation. In a case report recently published by Guadix et al., the authors showed the clinical benefit of LITT in a patient with Li-Fraumeni syndrome who had a recurrent glial tumor following prior surgical resection and adjuvant therapy [25]. They present LITT as a substitute for radiation therapy with relatively low risk for the patient. LITT has been shown to benefit patients suffering from tumor-related epilepsy as well. The results of seizure control have been shown to be comparable to open surgical resection in various adult series [26]. From a technical aspect, the use of LITT requires fixation of the guidance system to the skull using anchoring bolts, which can be problematic in the young age group when the skull is very thin. Some technical modifications have been discussed in the literature in a way of allowing the use of LITT in young children [27]. In many cases, it has been recommended to use the same trajectory for performing tumor biopsy before insertion of the laser catheter and performing the ablation. There is an associated complication rate of close to 13% [28]. The most commonly described adverse effect is periprocedural edema. In many cases, pre- and post-operative high-dose steroid treatment can help mitigate this phenomenon [29, 30].

## Emerging chemotherapy trials

While FUS and LITT present appealing avenues for minimally invasive ablation, emerging chemotherapy trials are exploring the role of new therapeutic regimens for pLGG. Chemotherapy has historically been utilized as a mainstay treatment for patients who require systemic therapy following surgery and has been first-line therapy for patients with unresectable tumors or progressive disease [31]. Commonly accepted first-line regimens include a combination of vincristine and carboplatin (VC); a combination of thioguanine, procarbazine, lomustine, and vincristine (TPCV); and vinblastine or carboplatin monotherapy [32–34]. Studies with these regimens have shown overall survival (OS) rates between 70 and 95% at 5 years, but progression-free survival rates (PFS) remain low at around 45–55% [31, 35]. Despite reasonable control rates, standard chemotherapeutic regimens have many associated toxicities, including myelosuppression, peripheral neuropathy, allergic reactions, sodium dysregulation, secondary malignancy, and hepatic or renal dysfunction. Approximately 50% of children will require an alternative second-line chemotherapy regimen given the associated toxicities and side effect profiles of these regimens [31].

Given the relatively low rates of tumor control associated with conventional first-line regimens, additional options are currently being explored. One ongoing phase II trial seeks to determine the efficacy of a combination of bevacizumab combined with vinblastine in chemotherapy-naïve patients harboring unresectable or progressive pLGG (NCT02840409). The results of this trial are not yet posted, and it is expected to conclude in 2026. Other ongoing trials seek to compare conventional chemotherapeutic regimens in combination with or in comparison to molecularly targeted therapies. For example, the PLGG–MEKTRIC trial seeks to compare the efficacy of MEK inhibitor trametinib with conventional chemotherapy in the form of mono-agent vinblastine (NCT05180825). Additionally, the LOGGIC/FIREFLY-2 trial (NCT05566795, EudraCT Number: 2022-001363-27), a phase 3 multicenter global randomized trial, is evaluating the efficacy, safety, and tolerability of tovorafenib, a pan-RAF inhibitor directed at MAPK activation signaling, compared to standard regimen combination CV or mono-agent vinblastine.

## Targeted therapy

The new era of molecular subtyping for tumors, adult and pediatric, holds promise for better treatment options for pediatric LGG. The current pathways that are being investigated are primarily MAPK/ERK and mTOR pathways. The main challenge now focuses on the efficacy of targeted therapy as either the initial treatment or as a second option when conventional treatment has failed, also examining the safety profile of the different treatment options and combinations and monitoring potential side effects. Despite encouraging responses to targeted therapy on neuroimaging, there is also a recognized rebound effect of tumor regrowth once the treatment is stopped [36, 37]. As such, it is imperative that patients treated with these agents remain on clinical protocols in which long-term side effects and efficacy can be documented. The use of targeted therapy as first-line therapy remains anecdotal, but preliminary results are promising [38].

In the mitogen-activated protein kinase/extracellular signal-regulated kinase (MAPK/ERK) pathway, a cascade of signal changes, including the activation of Ras, triggers the activity of the serine/threonine-protein kinase B-raf, which ultimately triggers mitogen-activated protein kinase kinase (MEK1and MEK2). Eventually, this leads to downstream activation of ERK, which then causes feedback inhibition of components at the beginning of the pathway [39]. Pediatric LGG often harbors alterations in the BRAF gene such as the known point mutation at V600E and the translocation of *KIAA1549*. These changes in the BRAF gene lead to alterations in the MAPK pathway promoting uncontrolled cell division and proliferation [40]. By understanding the changes found in the BRAF gene and the MAPK pathway, blockade of this aberrant pathway has become possible. Several studies have shown that having a pLGG with a BRAF mutation leads to significantly lower PFS as compared to the same tumor type absent this mutation (27% vs 60%), with more than a third of relapsed cases observed among patients that had GTR in their initial surgery [41, 42]. Moreover, it has been shown that pLGGs that harbor the V600E mutation have a much higher tendency to transform into HGG. One of the possible explanations is that, in 25% of the pLGG with BRAF^V600E^ mutations, there is also a deletion of CDKN2A. This may lead to tumor biology resembling HGG rather than LGG [43]. Whereas V600E mutated pLGG is usually found in supratentorial tumors, KIAA1549 fusion gene tumors are more commonly found in infratentorial tumors and have much better PFS and OS [39].

This has led to the use of BRAF inhibitors in pLGG treatment, as they can bind to mutated BRAF proteins and block the activation of MEK by inhibiting the MAPK/ERK pathway. In a recent series published by Leclair et al., 8 pediatric patients with pLGG were treated as first line with either a BRAF inhibitor (dabrafenib), a MEK inhibitor (trametinib), or the combination of the two [38]. The results showed satisfying efficacy and safety, but in several instances, the combination of these agents was required to achieve a favorable treatment outcome. Regarding the BRAF/MEK pathway, several trials have shown image response rates with varied results, such as 15% for trametinib in a study of recurrent disease or a 56% response rate with binimetinib (MEK162 inhibitor) in another publication [31, 44]. Recent studies testing the newer agent tovorafenib (2nd generation RAF inhibitor) have shown a 64% response rate for children with multiply recurrent pLGG. Children with neurofibromatosis type 1 (NF1) can normally be expected to develop low-grade gliomas in 10–15% of patients. Although it is quite common to find genetic alterations in the tumors affecting NF1 patients, very few of them are found to have BRAF variants [45]. Studies have shown that for this population who harbor NF1-associated tumors but who lack the BRAF alterations, the use of MEK inhibitors is very promising [46]. The major toxicities from the use of either MEK inhibitors or BRAF inhibitors are hair color changes, dermatologic complications, anemia, elevated creatinine phosphokinase (CPK), diarrhea, hypoalbuminemia, and more [44].

Another commonly altered pathway in pLGG is the mTOR pathway. This pathway includes a cascade of steps as well. The activation of the phosphatidylinositol 3-kinase/protein kinase B/mechanistic target of rapamycin (PI3K/AKT/mTOR) pathway eventually leads to activation of mTORC1/2, leading to cell survival, proliferation, and neoplasia [39]. Some data have suggested the potential benefit of mTOR inhibitors, such as everolimus, in the treatment of progressive pLGG [47]. This has led to efforts to characterize the benefit of combining mTOR inhibition with other inhibitory agents, such as trametinib in the trial of the PNOC (Pacific Pediatric Neuro-Oncology Consortium) for recurrent pLGG and pHGG. The major use of mTOR inhibitors has been for the treatment of tuberous sclerosis. The mTOR pathway was found to be very active in pLGG tumors associated with genetic syndromes such as NF1 and TS. Currently, many studies are ongoing, and with promising preliminary results. For TS patients, the use of everolimus has been shown to be very successful in treating subependymal giant cell astrocytomas (SEGAs) and has been proven useful for both tumor control and seizure control [48]. The adverse effects of the mTOR inhibitors have been well-characterized as relatively mild side effects, but certain populations can suffer from severe side effects as well [47].

More recently, trials have been initiated targeting tumors that harbor FGFR alterations. The fibroblast growth factor receptor (FGFR) family (FGFR1-4) is an important component in the MAPK pathway as well. The FGFR1 mutation is known to affect both the RAS/MAPK pathway as well as the PI3K/AKT/mTOR pathway; hence, there is great potential in controlling its activity [49]. One example is erdafitinib, used in the MATCH trial (Molecular Analysis for Therapy CHoice) of the Children’s Oncology Group (COG). Data has shown a partial response in more than half of the patients harboring pLGG and glioneural tumors. The possible toxicities for FGFR inhibitors are still being realized, with varied reports ranging from fingernail changes and hyperphosphatemia to more concerning reports of slipped capital femoral epiphyses [50].

The current trials of targeted therapies for pLGG include BRAF inhibitors, MEK inhibitors, mTOR inhibitors, and FGFR inhibitors. Other potential targets for LGG derive from the adult LGG studies but currently lack application to pediatric tumors. One example is the use of the IDH inhibitor vorasidenib, which has shown promising results in adults with LGG with PFS close to 2.5 times higher among the treated group vs placebo [51].

As mentioned, the main use of targeted therapy for pLGG is still for progressive or recurrent disease. The question of whether these should be the first-line treatment raises a number of ethical questions, such as the fiscal cost of these potentially life-long treatments compared to their efficacy, especially as compared to conventional systemic chemotherapy. The COG is currently running two prospective studies comparing selumetinib (MEK inhibitor) to conventional CV therapy (carboplatin/vincristine). Other groups across the world have suggested different paradigms comparing different MEK/BRAF inhibitors or other targeted therapeutic agents in comparison to the standard treatment. These include the combination of several agents such as dabrafenib and trametinib. One study worthy of mention is a study that led the Food and Drug Administration to approve the combination of dabrafenib and trametinib as first-line treatment for children 1 year of age and older with newly diagnosed BRAF^V600E^-mutant pLGG [44]. This study has shown that this combination led to a 47% response rate and 20.1 months of median PFS as compared to 11% and 7.4 months in the CV group, respectively. The results were promising both from an efficacy perspective and also from an adverse event profile, which was significantly better with the targeted therapy compared to the CV treatment. This, along with FDA approval, might be justification for oncologists to consider it as first-line therapy, although it is not clear if similar results can be achieved with single-agent treatment; hence, ongoing investigation is still necessary. The main criticism against the widespread use of targeted therapy agents in the Western world and even more so in developing countries is the significant increase in care costs, leading to an unbearable financial burden, especially in places where the cost of this expensive life-long treatment is borne by the family [52, 53]. In addition to the cost of the treatment, there is also the cost of pathologic analysis to consider. In recent work looking at the feasibility of sending samples for outside pathological consultation, it was shown in a cohort of 32 patients that the median turnaround time from the tumor samples’ shipment to the final report of the results was 23.5 days, with a median laboratory processing time of 16 days (range, 8–39 days) at a cost of US$1,000/sample. As the authors emphasized, it is important to include these data in the workup of these patients even in low-income countries. Ideally, these expenses should be covered by clinical research or compassionate use protocols rather than families [54].

## Discussion

Worldwide, the second most common cause of death in childhood is cancer [1]. With improvements in managing acute lymphoblastic leukemia, brain tumors are now the most common etiology of cancer-related mortality in childhood. With that in mind, LGG is the most common brain tumor in childhood, occurring in 30–40% of patients. When patients present with obstructive hydrocephalus, intractable seizures, or significant symptoms, the family is typically referred first for a neurosurgical opinion. It therefore behooves the neurosurgeon to engage in a multi-disciplinary team discussion regarding the optimal management of these patients.

Improvements in neuroimaging and image-guided resection now allow for more children to receive a surgical cure and with less surgical morbidity than ever before. In fact, in most experienced centers, death from brain tumor surgery is now a sentinel event. For those patients with diffusely infiltrative tumors, deep midline tumors, or those deemed unsafe for surgical resection, biopsy is also safer than ever before. Even with our diffuse brain stem tumors, complications from stereotactic biopsy are now less than 5% [55].

Rapid advances in the understanding of the tumor’s molecular biology have allowed for the development of a variety of novel treatment options. Although molecular profiling of tumor tissue is currently expensive and available mainly at reference centers, this technology is also advancing at a rapid pace. Whereas traditional histopathology has relied upon the subjective interpretation of what the pathologist sees when looking through the microscope, studies have shown that, even among the most experienced neuropathologists, there is significant discordance in the interpretation of what the pathologist sees [56]. With molecular profiling, this subjectivity is replaced by the objectivity of these newer technologies. As such, it is anticipated that over the next decade, molecular profiling will become faster and cheaper, the technology will become widely adopted, and molecular diagnosis will overshadow conventional histopathology. This underscores the importance of obtaining tissue for diagnostics whenever feasible.

The study of Wisoff et al., although a decade old, clearly demonstrated that in nearly half of patients with LGG, small amounts of residual tumor may remain stable on imaging for up to a decade following incomplete resection [2]. That said, we are all seeing children who are now 2–3 decades out from their original surgery in whom that small residual progresses. In most instances, new histology at the time of progression still mimics the original tumor, but the biology of the progression is not the same and can prove difficult to cure. Whereas in the past we would often dismiss patients with stable residual as “cured” after a decade of follow-up, we now know that they need lifelong clinical and radiographic monitoring. At the first sign of progression, new tissue should be obtained, and there should be multi-disciplinary discussion regarding when and which treatment is optimal.

Regarding low-grade chemotherapy, although response rates are high, it should be noted that many LGGs may naturally remain stable or may follow a very indolent course. Studies noting “failure to progress” over a 3–5-year period may, in some instances, be recapitulating the natural history of the disease. In the long run, the chances of conventional chemotherapy “curing” a low-grade glioma are less than 10%. Radiation therapy, on the other hand, has a higher likelihood of cure but raises concern in a slow-growing, indolent process for the resultant malignant progression or secondary neoplasia. With more focal radiation techniques, such risks seem to be diminished. In a recent review of 101 children treated for posterior fossa ependymoma before the age of 3 years with surgical resection followed by 3-D conformal radiotherapy, at 25 years follow-up, only 6 children developed malignant progression or secondary neoplasia, whereas 24 children in the series had died of progression of their primary tumor [57].

Targeted therapies, more than immunotherapy trials, have shown promising results in selected individuals. Anecdotal cases of significant responses to these agents in children who were otherwise deemed incurable have become commonplace. For instance, multiple studies have shown dramatic responses in high-grade and recurrent low-grade gliomas with BRAF^V600E^ mutations treated with vemurafenib when all other standard therapies have failed. Given the poor response to conventional chemotherapy and radiation for children with gangliogliomas of the brain stem which harbor V600e mutations, vemurafenib is now being proposed as first-line therapy for these patients [58, 59]. However, though study data gives us a picture of what percentage of patients may respond to treatment, we have a poor understanding of what the duration of the response will be and how to predict who will stop responding over time. Furthermore, two-thirds of children on these agents will suffer grade III toxicities which may lead to a discontinuation of the drug and subsequent rebound of tumor re-growth. Finally, these agents are prohibitively expensive considering that a young child will likely be on the drug lifelong. One treatment paradigm currently under study for adults with papillary craniopharyngioma is to treat with targeted agents until maximal tumor response is seen, then apply focal radiotherapy with discontinuation of the drugs [60]. At present, long-term results for this approach are pending. Nonetheless, it should be stated that patients being treated with targeted therapies, laser ablation, focused ultrasound, or other emerging technologies should all be enrolled in clinical protocols in which they are carefully followed.

## Conclusions

For most patients diagnosed with low-grade gliomas, the chances of being alive a decade later are over 80% [2]. At present, for those amenable to maximal safe surgical resection, this is still the best opportunity for cure. Rapid advances in both neuroimaging and molecular profiling have led to the development of promising treatment alternatives for patients with surgically incurable low-grade glioma such as optic pathway gliomas, diffuse cortical tumors, or those with diffuse midline tumors. The molecular profiling of individual tumors offers information that is more objective, more predictive, and more precise than traditional histopathology, making the importance of obtaining tumor tissue in each patient with this disease of critical importance. Although there may come a day when patients with low-grade glioma can be diagnosed and treated based upon imaging alone or biopsy only, currently, the management of these tumors remains primarily neurosurgical within the confines of a multi-disciplinary team.

## Data Availability

No datasets were generated or analyzed during the current study.
